# Cross-sectional study of the proportion of antibiotic use during childbirth in full-term deliveries in Finland

**DOI:** 10.1186/s12884-023-05368-0

**Published:** 2023-01-21

**Authors:** Susanna Gardemeister, Kirsi Skogberg, Terhi Saisto, Anne Salonen, Willem M. de Vos, Katri Korpela, Kaija-Leena Kolho

**Affiliations:** 1 Children’s Hospital, University of Helsinki, Stenbäckinkatu 11, FI-00029 HUS Helsinki, Finland; 2 Division of Infectious Diseases, Inflammation Centre, Helsinki University Hospital, and University of Helsinki, Helsinki, Finland; 3 Department of Obstetrics and Gynaecology, Helsinki University Hospital, HUS, and University of Helsinki, Helsinki, Finland; 4 Human Microbiome Research Program, Faculty of Medicine, University of Helsinki, Helsinki, Finland; 5 Laboratory of Microbiology, Wageningen University, Wageningen, The Netherlands; 6 Faculty of Medicine and Health Technology, Tampere University, Tampere, Finland

**Keywords:** Antibiotic prophylaxis, Caesarean section, Group B Streptococcus, Infant, Pregnancy

## Abstract

**Purpose:**

In developed countries, data on the frequency of antibiotics given to mothers during childbirth are limited beyond the overall effect of all various prophylactic indications. Also, data on the impact of such antibiotics to the well-being of term babies are scarce. We aimed to characterize the frequency of antibiotic use during childbirth of term pregnancy. Secondly, we assessed whether the use of antibiotics was associated with any symptoms in infants.

**Methods:**

This was a cross-sectional study of 1019 term deliveries of women participating in the prospective Health and Early Life Microbiota (HELMi) birth cohort study between March 2016 and March 2018 in the capital region of Finland. The data on antibiotic use were collected from the hospital records.

**Results:**

In total, 37% of the mothers received antibiotics during childbirth and 100% in Caesarean Sects. (17% of the deliveries). Less than 5% of antibiotics were non-prophylactic. In vaginal deliveries, the most common indication (18%) was prophylaxis for Group B Streptococcus. The most frequently used antibiotics were cefuroxime (22%) and benzylpenicillin (15%), and 56% received only one dose. In infants exposed to antibiotics during delivery, defecation frequency was higher during the first months (*p*-value < 0.0001- 0.0145), and weight gain was higher at the age of three months (*p*-value 0.0371).

**Conclusion:**

More than every third new-born in a developed country is exposed to antibiotics during birth. Our findings support the hypothesis that maternal antibiotics given during birth have an impact on the well-being of the infants. These findings should inform current policies for prophylactic antibiotics in childbirth.

## Introduction

During the first years of life, the intestinal microbiota of infant develops radically. The most common factors that disturb the development of the infant microbiota are caesarean section (CS) and the use of antibiotics during pregnancy and delivery as well as in infancy [1–3]. Prophylactic antibiotics are commonly used during delivery and the indications have expanded during the recent years. The primary aims of prophylaxis are to prevent neonatal group B streptococcus (GBS) early-onset disease and postpartum infections, especially after CS. Approximately 18% of women are colonized with GBS worldwide [4] but the strategies to identify mothers to whom intrapartum GBS prophylaxis is recommended vary. The most common are risk factor-based assessment, rectovaginal culture at 36 0/7–37 6/7 weeks of gestation and point-of-care polymerase chain reaction (PCR) screening on admission [5, 6].

However, antibiotics during delivery do not only decrease the vertical transmission of pathogenic microbes from mother to child but also that of commensals, and correspondingly increase the relative number of microbes originating from the environment, for example from other patients in the same ward in the hospital [7]. It has been reported that those new-borns who have been exposed to antibiotics during delivery or postnatally have lower amount of *Bifidobacterium* and *Bacteroides* spp. in their gut microbiome than others [8, 9]. It is noticeable that the development of the infant microbiome follows certain time frames and when disrupted by antibiotics the natural developmental pattern is disrupted [10].

The long-term consequences of an antibiotic course for the infant’s health depend on the timing. For example, prenatal antibiotics seem to increase the risk for very early-onset inflammatory bowel disease [11] and childhood asthma [12] whereas the antibiotic courses of infancy have been associated with childhood obesity [13, 14]. It has been noted that antibiotics have different capabilities to pass through the placenta [15]. Furthermore, the drug delivery may vary according to gestational age and presence of infection, for example, chorioamnionitis [15].

The purpose of this study was to research in a cross-sectional study to what extent mothers receive antibiotics during term delivery in a developed country. The other aim was to find out whether there was an association between such antibiotics on child’s health and weight gain during the first months. We utilized the data from southern Finland collected for the prospective study HELMi [16] monitoring infant health focusing here on the use of maternal antibiotics during delivery.

## Materials and methods

### Data collection

This study is a part of the Health and Early Life Microbiota (HELMi) study [16] which is a longitudinal Finnish general population birth cohort that consist of a little over one thousand children and their families, mainly from the capital region of Finland. The purpose of the HELMi study is to research possible associations between the intestinal microbiota development, environmental and genetic factors as well as health outcomes [16]. All infants in the cohort were born full term in 2016–2018 in public healthcare. In Finland, most babies are born in public hospitals and home births are rare. At 3 months of age, 86% of the babies were exclusively breastfed, 2% exclusively formula-fed and 70% were given probiotic products [16].

The data about antibiotics during delivery were collected from the maternity records of the Hospital District of Helsinki and Uusimaa (HUS). The data of the HELMi study were prospectively collected using extensive online questionnaires focusing on infant health at weekly to monthly intervals [16]. In addition, the families attended study visit at 3 months (participation rate 95%) [16]. Here we utilized data on defecation frequency, stool consistency, crying, childhood infections and weight during the first months.

### Ethics

The families that participated in the HELMi study have given their written informed consent, and the study has been approved by the ethical committee of HUS (HUS 1797/2016). At least one parent from each family needed to understand Finnish to be able to fill in the questionnaires. Higher educated parents were slightly over-represented, as well as parents who had allergy or other immune-mediated diseases [16].

### Prophylaxis policy during the study period

At the time of the study, all women in delivery were screened routinely for GBS with a PCR-based test at the time of admission according to local hospital guidelines. Proven or unclear GBS carriage status of a symptomless mother, urinary tract infection during pregnancy caused by GBS, former GBS early-onset disease of the mother or of a previous child indicated intrapartum intravenous prophylactic course of penicillin. In case of mother`s penicillin allergy with low risk of anaphylaxis, clindamycin, erythromycin, or cefuroxime were used, and with high risk of anaphylaxis, vancomycin. If the mother had a concomitant infection, antibiotic was chosen to cover both indications. Typical prophylaxis in CSs included one dose of cefuroxime at the beginning of surgery or in case of allergy to cefuroxime, one dose of clindamycin or vancomycin, respectively. Cefuroxime was also indicated in case there had been preterm pre-labour rupture of membranes (PPROM).

There are minor differences in the types of prophylactic antibiotics recommended in various countries. For example, in the United States (US) first-generation cephalosporins such as cefazolin are recommended in cases of mother`s penicillin allergy with low risk of anaphylaxis, and in high risk for anaphylaxis either clindamycin, or vancomycin. In CSs there are more diverse practices as in the US cefazolin is recommended for antibiotic prophylaxis with the addition of adjunctive azithromycin prophylaxis [17].

### Statistical analysis

Data are presented as median and interquartile range (IQR) unless otherwise stated. The statistical comparisons were made with the non-parametric Mann–Whitney U test and the Chi square test when appropriate using Graph Pad Prism -program (version 9.2.0). The value of statistical significance was *p* < 0.05.

## Results

### Study population

Data were collected from 1019 deliveries occurring between March 2016 and March 2018 in the Finnish public healthcare region HUS covering the capital region. All except five births occurred in a hospital. The infants were born full term at gestational weeks 37–42 without known congenital defects and 51.4% of the infants were males. The median time of discharge from the hospital was 2 days after birth (IQR 2–3).

The age range of the mothers was from 15 to 45 years while the median age was 33 years (IQR 30–36; data missing in 15 cases). The median pre-pregnancy BMI was 23.4 kg/m^2^ (IQR 20.9–24.9 kg/m^2^; data missing in 23 cases).

### Proportion and indications of intrapartum antibiotic use

Of all deliveries, 83.1% were vaginal and 16.9% CSs (Table 1). Of CSs, 55.2% were performed on emergency and of vaginal births, 20.7% were induced. Of the vaginal deliveries, 23.7% of the mothers received antibiotics, but in the induced vaginal deliveries the frequency was significantly higher (40.6%; *p*-value < 0.0001). All mothers who gave birth by CS received antibiotics. At least one antibiotic was received by 36.6% of all mothers.

**Table 1:** Prophylactic and non-prophylactic antibiotic use during 1019 full-term deliveries

	Antibiotics	Prophylactic	Non-prophylactic	Indication unclear
All deliveries *n* = 1 019	373 (36.6%)	353 (34.6%)	49 (4.8%)	1 (0.1%)
Vaginal deliveries *n* = 847	201 (23.7%)	181 (21.4%)	33 (3.9%)	1 (0.1%)
*Spontaneous vaginal deliveries* *n* = *672*	130 (19.3%)	120 (17.9%)	18 (2.7%)	1 (0.2%)
*Induced vaginal deliveries* *n* = *175*	71 (40.6%)	61 (34.9%)	15 (8.6%)	0 (0.0%)
Caesarean sections *n* = 172	172 (100.0%)	172 (100.0%)	16 (9.3%)	0 (0.0%)
*Elective caesarean sections* *n* = *77*	77 (100.0%)	77 (100.0%)	2 (2.6%)	0 (0.0%)
*Emergency caesarean sections* *n* = *95*	95 (100.0%)	95 (100.0%)	14 (14.7%)	0 (0.0%)

The pre-pregnancy BMI of the mothers who gave birth by CS was significantly higher compared to mothers giving vaginal birth (the median 24.0 kg/m2 (IQR 21.9–26.4) and 22.5 kg/m^2^ (IQR 20.8–24.6), respectively; *p*-value < 0.0001). There was no statistically significant association between pre-pregnancy BMI and whether vaginal delivery was induced or not, or BMI and the urgency of CS (*p*-values 0.0651–0.2381). Furthermore, when only vaginal births were considered, there was no association between BMI and receiving antibiotic during delivery (*p*-value 0.4125).

For prophylactic indications, antibiotics were used in 34.6% of all deliveries. The most common prophylactic indications were proven or unclear GBS carriage status of the mother (17.1% of all deliveries) and CS. Other prophylactic indications were PPROM, covering 2.4% of all deliveries, Foley catheter induction, 1.3%, and green amniotic fluid, 0.1% (Tables 1 and 2).

**Table 2:** Antibiotic use by indication during 1019 full-term deliveries

	Indications to antibiotic use				
Prophylactic antibiotics *n* = 353^a,b^	Proven or unclear GBS^c^ carriage status 174 (49.3%)	Caesarean section 172 (49.7%)	Preterm pre-labour rupture of membranes 24 (6.8%)	Foley catheter induction 13 (3.7%)	Green amniotic fluid 1 (0.3%)
Non-prophylactic antibiotics *n* = 49	Signs of chorioamnionitis 46 (93.9%)	Infection unrelated to delivery 4 (8.2%)			

^a^One indication stayed unclear

^b^66 antibiotic courses include more than one indication

^c^Group B streptococcus

For non-prophylactic indications, antibiotics were used in 4.8% of all deliveries. Signs of chorioamnionitis such as raised temperature, fever and elevated leucocyte levels or C-reactive protein (CRP) covered 4.5% of all deliveries, while infections unrelated to delivery, such as urinary tract or ear infection, covered the rest. The indication of one antibiotic course was unclear (Tables 1 and 2).

Most frequently used antibiotic was cefuroxime since it was received by 22.3% of all mothers and 95.3% of mothers who gave birth by CS. Benzylpenicillin was received by 15.4% of all mothers and it was most frequently used in induced vaginal deliveries. Metronidazole was received by 2.9% and clindamycin by 1.4% off all mothers and they were most frequently used in emergency CSs. In addition, one mother received nitrofurantoin (Table 3). Both oral agents and intravenous antibiotics were included.

**Table 3:** Use of different types of antibiotics during 1019 full-term deliveries

	Benzylpenicillin	Cefuroxime	Metronidazole	Clindamycin	Nitrofurantoin
All deliveries *n* = 1 019	157 (15.4%)^a^	227 (22.3%)	30 (2.9%)	14 (1.4%)	1 (0.1%)
Vaginal deliveries *n* = 847	140 (16.5%)	63 (7.4%)	12 (1.4%)	7 (0.8%)	1 (0.1%)
*Spontaneous vaginal deliveries* *n* = *672*	97 (14.4%)	31 (4.6%)	7 (1.0%)	6 (0.9%)	1 (0.2%)
*Inducted vaginal deliveries* *n* = *175*	43 (24.6%)	32 (18.3%)	5 (2.9%)	1 (0.6%)	0 (0.0%)
Caesarean sections *n* = 172	17 (9.9%)	164 (95.3%)	18 (10.5%)	7 (4.1%)	0 (0.0%)
*Elective caesarean sections* *n* = *77*	0 (0.0%)	74 (96.1%)	3 (3.9%)	2 (2.6%)	0 (0.0%)
*Emergency* *caesarean sections* *n* = *95*	17 (17.9%)	90 (94.7%)	15 (15.8%)	5 (5.3%)	0 (0.0%)

^a^48 mothers received several different antibiotics and each of them have been recorded

Mothers received a median of one dosage (IQR 1–3) of antibiotics. Among vaginal deliveries, the median dosage was 2 (1–3) and among CSs 1 (1–1).

### Gastrointestinal function and crying of the babies

**Fig. 1:**
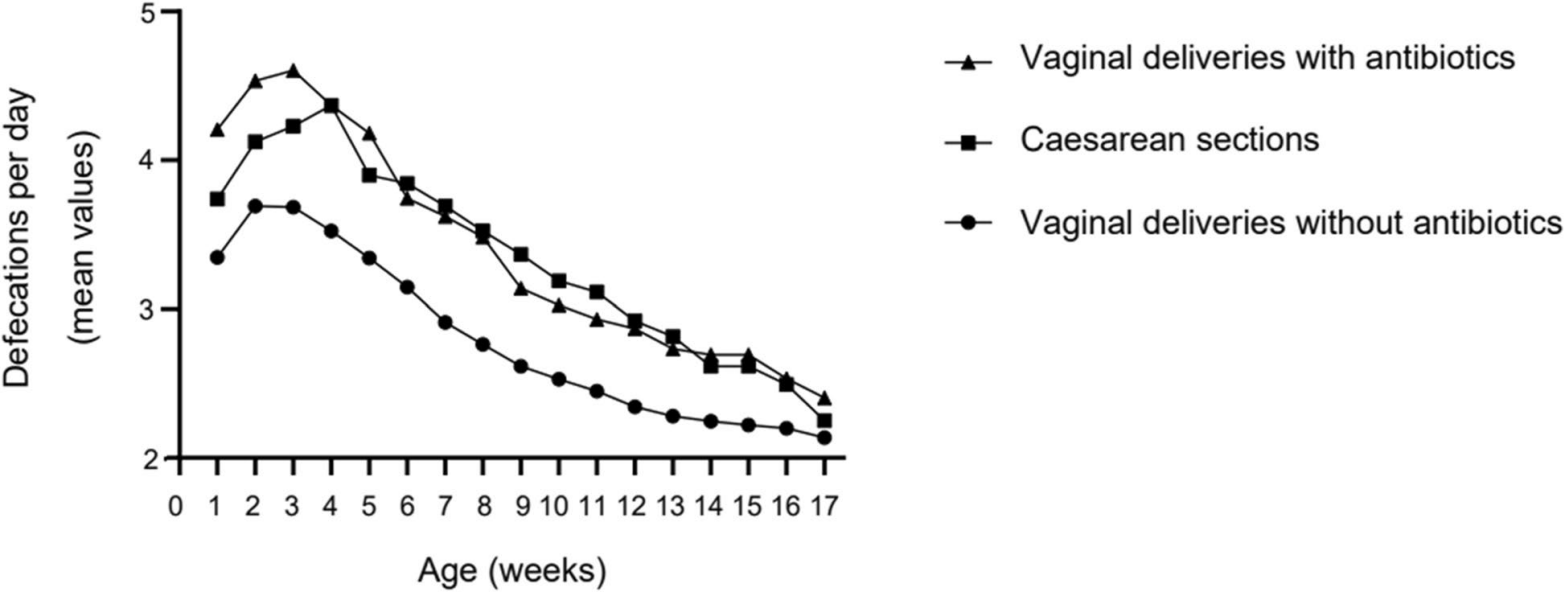
Defecation frequency of 1019 babies born full-term

The defecation frequency of the infant was statistically significantly higher if the mother had received intrapartum antibiotics when compared to those infants whose mothers had not received antibiotics (during the first week, the median defecation frequency 3.2 per day (IQR 2.8–4.0) and 2.6 (IQR 2.3–3.3), respectively; *p*-value < 0.0001). The association stayed statistically significant during the entire 17 weeks observation period (*p*-value < 0.0001- 0.0145) (Fig. 1).

If the mother had received antibiotics, the defecation frequency was higher among vaginally born infants comparing to infants born by CS (during the first week, the median 4.0 per day (IQR 2.9–5.7) and 3.6 (IQR 2.9–5.0), respectively; *p*-value 0.0425). The association stayed significant during the first three weeks (*p*-value 0.0250–0.0425) (Fig. 1).

When observing all deliveries, there was no association between defecation frequency and birth mode (during the first week, among CSs the median 3.6 per day (IQR 2.3–5.0) and among vaginal deliveries 3.0 (IQR 2.0–5.0)). Furthermore, there was no statistically significant association between intrapartum antibiotics and the stool consistency**,** or the amount of crying in the infants as reported online by their parents during the first 17 weeks (whether infants were exposed to antibiotics, during the first week the median stool consistency of infants was 6 at the Bristol stool chart (IQR 6–6) and estimated amount of crying 1 h per day (IQR 1–1)).

### Weight gain of the babies

At the age of three months, the weight of the children that had been exposed to intrapartum antibiotics was statistically higher than the weight of those children who had not been exposed to antibiotics (the median 6.4 kg (IQR 5.9–6.9) and 6.3 kg (IQR 5.7–6.8), respectively; *p*-value 0.0371). The difference in weight was not significant during the previous months, and it disappeared by the age of four months. There was no significant weight difference between children born by vaginal delivery versus CS at the age of three months.

### Infections of the infants during follow up

During the first four months, 53.1% of the infants got an infection (45.6% respiratory tract, 1.2% gastrointestinal, 0.3% urinary tract and 6.0% other infection). There was no statistically significant association between the use of intrapartum antibiotics and the incidence of infections during that time (during the first month, 14.7% of those infants who were exposed to antibiotics got an infection, whereas 21.5% of those infants that were not exposed).

## Discussion

The purpose of this study was to find out the frequency of antibiotic use during term deliveries in a developed country as well as the indications and types of antibiotics used in a comprehensive study cohort. The other aim was to find out whether the antibiotic use is associated with infant health during the first three months of life. The data about antibiotics during delivery were collected from the maternity records and combined with the data from the online survey of the participants of the prospective HELMi cohort [16]. All 1019 infants were born between March 2016 and March 2018 at gestational weeks 37—42 without known congenital defects.

Overall, antibiotics were used during 36.6% of all deliveries. The number is slightly larger than in a corresponding Danish study including 706 women, where the frequency was 33% [18] and slightly smaller comparing to frequency in the US, > 40% [19]. In our study, 23.7% of the mothers who gave birth vaginally received antibiotics. Comparable to that, in a Canadian cohort study including 198 infants, 27% of vaginally born infants were exposed to intrapartum antibiotics mostly due to GBS prophylaxis or PPROM [20]. In our cohort, the indication for antibiotics was non-prophylactic in less than 5% of the deliveries.

In our cohort, all mothers with intended vaginal delivery were screened routinely for GBS with PCR-based test at the time of admission to delivery ward, and 17% of all mothers received antibiotics for proven or unclear GBS carriage status. The number is equivalent to a worldwide amount of GBS carriers (18%) [4]. GBS screening policy is heterogeneous [5]. In a study covering 95 countries, approximately two thirds of countries reported a GBS screening policy, most often based either on rectovaginal culture or clinical risk factors and only few reported point-of care PCR-screening [5]. In maternity hospitals in Helsinki and Uusimaa district PCR-screening performed by midwives by point of care test is used, since it proves rapid information of the current GBS status of the mother, is easy to perform and effective in preventing GBS early-onset disease in infants (0.15 cases during first 72 h/1000 live births between years 2016 and 2017). While discussing the potential side effects of antibiotics, it should be acknowledged that intrapartum antibiotic prophylaxis is the only effective treatment so far to prevent GBS early-onset disease and therefore effective screening at the time of delivery is necessary [21].

All mothers who gave birth by CS received antibiotics as prophylaxis due to birth mode as recommended in international guidelines, since prophylaxis reduces the incidence of maternal wound infections and endometritis [22, 23]. The effect is especially profound in women undergoing emergency CS and lower in non-laboring women with intact membranes undergoing elective CS [23]. In our study, CSs covered 16.9% of the deliveries corresponding to the national rate of 16.7% in Finland [24]. This is a slightly smaller number than a corresponding recent number of Northern Europe 22.4%, worldwide percentage being 18.6% but with large country-specific variations [25]. The proportion of CS, however, has been on the rise during the last decades [25]. Reasons for the phenomenon are complex, varying from maternal characteristics to ethical considerations [26]. As a result, an increasing number of infants are exposed to antibiotics during delivery [19].

Administration of prophylactic antibiotics after the cord is clamped can prevent infant´s exposure to antibiotics during CS. However, current WHO recommendation is to administer prophylactic antibiotic before surgical incision based on meta-analyses concluding that this policy reduces the risk of surgery [27]. On the contrary, recent large Swiss study did not confirm this finding [28]. At the time of our study, local hospital guidelines instructed to start prophylactic antibiotics 30–60 min before incision.

In our study, higher pre-pregnancy BMI of the mother was statistically significantly associated with a higher frequency of CS, and therefore also the use of intrapartum prophylactic antibiotics. Other studies have shown that delivery progresses more slowly when maternal BMI increases [29, 30], leading more frequently to emergency CSs.

Intriguingly, the antibiotic use during delivery was associated with gastrointestinal function of the infant. Defecation frequency of the infant during the 17-week follow-up period was increased significantly when the mother had received intrapartum antibiotics. The difference may not be clinically relevant, but supports the hypothesis that antibiotics used during delivery affect the gastrointestinal tract and infant microbiota [31, 32]. During the first three weeks, those infants who received antibiotics during vaginal delivery had higher defecation frequency comparing to infants born by CS, which may indicate that antibiotics received just before birth during CS may not modify the infant microbiome as strongly as antibiotics received earlier. The finding may also be explained by different the types of antibiotics used during delivery since benzylpenicillin was more frequently used in vaginal deliveries and cefuroxime in CSs. In addition, the median dosage of antibiotics during delivery was 2 among vaginal deliveries and 1 among CSs supporting the result. Importantly, intrapartum antibiotics did not associate with greater amount of crying or did not carry along an increased risk for impaired health of the infant during the first 17 weeks.

Earlier studies of the association between the growth of the infant and antibiotics during pregnancy and early age have reported that there is some variation between different antibiotics. For example, especially macrolides and beta-lactams such as penicillin associated with a greater weight gain during infancy and childhood [33–35]. This may be explained by variable transplacental transmission of different antibiotics. It also seems that the timing of the antibiotic course is of major importance. For example, one study reported that antibiotics taken during the first week of life were associated with smaller growth whereas the later use of antibiotics during the first year was associated with a greater growth and weight gain [36].

We observed a positive association between intrapartum antibiotics and the weight of the infant at the age of three months. The infants who had been exposed to antibiotics during delivery weighted on average 140 g more than the others and the finding was not explained by the birth mode. Although the weight difference disappeared by four months the results are in line with previous studies suggesting that the antibiotics increase weight gain in children most likely through modification of the intestinal microbiota [35].

### Strengths and limitations

To our knowledge, this study cohort of term pregnancies included in the analyses of antibiotic use is one of the largest reported. This cohort represents well the general population as the number of CSs (16.9%) corresponded accurately to the number of CSs in Finland (16.7% in 2017 [24]). Also, the data were comprehensive and reliable, collected from one hospital district with uniform patient charts and clinical practice. Notably, the data on infant health was prospectively collected on weekly online questionnaires covering the 17-week observation period [16]. As a limitation, we did not have the data on infant heights at three months of age. Importantly, there were no major health concerns related to infant health when being exposed to antibiotics during delivery.

As another limitation, there was slight uncertainty of the data recorded in patient charts of a few mothers. In such cases, the entire maternity record was thoroughly reviewed. However, missing data were rare and occasional unclear markings did not affect the main results.

## Conclusions

This study shows that the use of antibiotics during term delivery was astonishingly frequent since more than every third new-born was exposed to intrapartum antibiotics. In most cases, the indication for antibiotic use was prophylactic, either a positive test results of the mother in GBS-screening or CS. However, antibiotics are not fully harmless as they modify the microbiota of the mother and the new-born. Reassuringly, we did not observe any major immediate health issues in infants exposed to antibiotics during delivery, but long-term effects need to be addressed.

Prophylaxis policy, type of antibiotics used, and screening techniques vary between countries and therefore the most optimal prophylactic use of antibiotics should be investigated to optimize the benefits and minimize the harms to mother and infant and the risk of increasing antimicrobial resistance. Also, further understanding is needed on how to enhance the recovery of the gut microbiota after antibiotics.

## Data Availability

All data generated or analysed during this study are included in this published article.

## Abbreviations

CS: Caesarean section
CRP: C-reactive protein
GBS: Group B streptococcus
HELMi: Health and Early Life Microbiota cohort
HUS: Hospital District of Helsinki and Uusimaa
IQR: Interquartile range
PCR: Polymerase chain reaction
PPROM: Preterm pre-labour rupture of membranes
US: United States

## References

[1] Shao Y, Forster SC, Tsaliki E, Vervier K, Strang A, Simpson N (2019). Stunted microbiota and opportunistic pathogen colonization in caesarean-section birth. Nature.

[2] Wampach L, Heintz-Buschart A, Fritz JV, Ramiro-Garcia J, Habier J, Herold M (2018). Birth mode is associated with earliest strain-conferred gut microbiome functions and immunostimulatory potential. Nat Commun.

[3] Korpela K, de Vos WM (2018). Early life colonization of the human gut: microbes matter everywhere. Curr Opin Microbiol.

[4] Russell NJ, Seale AC, O’Driscoll M, O’Sullivan C, Bianchi-Jassir F, Gonzalez-Guarin J (2017). Maternal Colonization with Group B Streptococcus and Serotype Distribution Worldwide: Systematic Review and Meta-analyses. Clin Infect Dis.

[5] le Doare K, O’Driscoll M, Turner K, Seedat F, Russell NJ, Seale AC (2017). Intrapartum Antibiotic Chemoprophylaxis Policies for the Prevention of Group B Streptococcal Disease Worldwide: Systematic Review. Clin Infect Dis.

[6] Prevention of Group B Streptococcal Early-Onset Disease in Newborns. ACOG Committee Opinion, Number 797. Obstet Gynecol. 2020;135:e52–71. 10.1097/AOG.0000000000003668 31977795

[7] Li W, Tapiainen T, Brinkac L, Lorenzi HA, Moncera K, Tejesvi MV (2021). Vertical Transmission of Gut Microbiome and Antimicrobial Resistance Genes in Infants Exposed to Antibiotics at Birth. J Infect Dis.

[8] Tapiainen T, Koivusaari P, Brinkac L, Lorenzi HA, Salo J, Renko M (2019). Impact of intrapartum and postnatal antibiotics on the gut microbiome and emergence of antimicrobial resistance in infants. Sci Rep.

[9] Princisval L, Rebelo F, Williams BL, Coimbra AC, Crovesy L, Ferreira AL (2021). Association Between the Mode of Delivery and Infant Gut Microbiota Composition Up to 6 Months of Age: A Systematic Literature Review Considering the Role of Breastfeeding. Nutr Rev.

[10] Korpela K, Salonen A, Saxen H, Nikkonen A, Peltola V, Jaakkola T (2020). Antibiotics in early life associate with specific gut microbiota signatures in a prospective longitudinal infant cohort. Pediatr Res.

[11] Örtqvist AK, Lundholm C, Halfvarson J, Ludvigsson JF, Almqvist C (2019). Fetal and early life antibiotics exposure and very early onset inflammatory bowel disease: a population-based study. Gut.

[12] Bai L, Zhao D, Cheng Q, Zhang Y, Wang S, Zhang H (2019). Trimester-specific association between antibiotics exposure during pregnancy and childhood asthma or wheeze: the role of confounding. Ann Epidemiol.

[13] Cox LM, Blaser MJ (2015). Antibiotics in early life and obesity. Nat Rev Endocrinol.

[14] Turta O, Rautava S (2016). Antibiotics, obesity and the link to microbes - what are we doing to our children?. BMC Med.

[15] Syme MR, Paxton JW, Keelan JA (2004). Drug transfer and metabolism by the human placenta. Clin Pharmacokinet.

[16] Korpela K, Dikareva E, Hanski E, Kolho KL, de Vos WM, Salonen A (2019). Cohort profile: Finnish Health and Early Life Microbiota (HELMi) longitudinal birth cohort. BMJ Open.

[17] Committee on Practice Bulletins-Obstetrics.ACOG Practice Bulletin No. 199: Use of Prophylactic Antibiotics in Labor and Delivery. Obstet Gynecol. 2018;132:e103–19. 10.1097/AOG.0000000000002833 30134425

[18] Stokholm J, Schjørring S, Pedersen L, Bischoff AL, Følsgaard N, Carson CG (2013). Prevalence and predictors of antibiotic administration during pregnancy and birth. PLoS One.

[19] Ledger WJ, Blaser MJ (2013). Are we using too many antibiotics during pregnancy?. BJOG.

[20] Hughes BL (2016). Antibiotic prophylaxis in pregnancy - Benefit without harm?. BJOG.

[21] Rao GG, Khanna P (2020). To screen or not to screen women for Group B Streptococcus (Streptococcus agalactiae) to prevent early onset sepsis in newborns: recent advances in the unresolved debate. Ther Adv Infect Dis.

[22] van Schalkwyk J, van Eyk N, Yudin MH, Boucher M, Cormier B, Gruslin A, et al. Antibiotic Prophylaxis in Obstetric Procedures.J Obstet Gynaecol Can. 2010;32:878–84. 10.1016/S1701-2163(16)34662-X PMC7128122 21050523

[23] Smaill FM, Grivell RM (2014). Antibiotic prophylaxis versus no prophylaxis for preventing infection after cesarean section. Cochrane Database Syst Rev.

[24] Perinatal statistics - parturients, deliveries and newborns. Official Statistics of Finland (OSoF). http://www.stat.fi/til/sysyvasy/index_en.html. Accessed 8 Dec 2022.

[25] Betrán AP, Ye J, Moller AB, Zhang J, Gülmezoglu AM, Torloni MR (2016). The increasing trend in caesarean section rates: Global, regional and national estimates: 1990–2014. PLoS One.

[26] Linton A, Peterson MR, Williams TV (2004). Effects of Maternal Characteristics on Cesarean Delivery Rates among U.S. Department of Defense Healthcare Beneficiaries, 1996–2002. Birth.

[27] WHO recommendation on Prophylactic antibiotics for women undergoing caesarean section. Geneva: World Health Organization; 2021. 34185445

[28] Sommerstein R, Marschall J, Atkinson A, Surbek D, Dominguez-Bello MG, Troillet N (2020). Antimicrobial prophylaxis administration after umbilical cord clamping in cesarean section and the risk of surgical site infection: a cohort study with 55,901 patients. Antimicrob Resist Infect Control.

[29] Shenouda C, Wijesooriya A, Toufeili A, Miller MR, Penava D, de Vrijer B (2020). Labour Progression in Obese Women: Are Women With Increased Body Mass Index Having Unnecessary Cesarean Sections?. J Obstet Gynaecol Can.

[30] Ellis JA, Brown CM, Barger B, Carlson NS (2019). Influence of maternal obesity on labor induction: a systematic review and meta-analysis. J Midwifery Women’s Health.

[31] Zimmermann P, Curtis N (2020). Effect of intrapartum antibiotics on the intestinal microbiota of infants: a systematic review. Arch Dis Child Fetal Neonatal Ed.

[32] Dierikx TH, Visser DH, Benninga MA, van Kaam AHLC, de Boer NKH, de Vries R (2020). The influence of prenatal and intrapartum antibiotics on intestinal microbiota colonisation in infants: a systematic review. J Infect.

[33] Mbakwa CA, Scheres L, Penders J, Mommers M, Thijs C, Arts ICW (2016). Early life antibiotic exposure and weight development in children. J Pediatr.

[34] Zhao Y, Zhou Y, Zhu Q, Xia B, Ma W, Xiao X (2019). Determination of antibiotic concentration in meconium and its association with fetal growth and development. Environ Int.

[35] Saari A, Virta LJ, Sankilampi U, Dunkel L, Saxen H (2015). Antibiotic exposure in infancy and risk of being overweight in the first 24 months of life. Pediatrics.

[36] Kamphorst K, Oosterloo BC, Vlieger AM, Rutten NB, Bunkers CM, Wit EC (2019). Antibiotic Treatment in the First Week of Life Impacts the Growth Trajectory in the First Year of Life in Term Infants. J Pediatr Gastroenterol Nutr.

